# Supplementary material for: Highly selective self-condensation of cyclohexanone: the distinct catalytic behaviour of HRF5015
Source: R Soc Open Sci. 2020 Oct 7;7(10):200123. doi: 10.1098/rsos.200123 (PMC7657892; doi:10.1098/rsos.200123)
Supplement: Peng et.al_ figures_ESM.doc [file rsos200123supp1.docx]

Highly selective self-condensation of cyclohexanone: the distinct catalytic behavior of HRF5015

Xiujing Peng ^a,b^, Shah Zeb ^a,b^, Jianguo Zhao ^a^, Miaomiao Zhang ^a^, Yu Cui ^a^, Guoxin Sun* ^a,b^

^a^.School of Chemistry and Chemical Engineering, University of Jinan, 250022, Jinan, China.

^b^.Institute for Smart Materials & Engineering, University of Jinan, 250022, Jinan, China.

Abstract

HRF5015, a perfluorosulfonic acid resin catalyst with unique pore structures, was investigated in the catalytic self-condensation of cyclohexanone under mild conditions. The morphology of HRF5015 was characterized by transmission electron microscopy (TEM) and atomic force microscope (AFM), and the reaction mechanism was studied by in-situ diffuse reflectance infrared fourier transform spectroscopy (DRIFTS). The effects of reaction time and temperature on the yield of dimer were investigated under nitrogen atmosphere. The results show that the reaction temperature is low and especially, the selectivity of dimer is close to 100%. The apparent activation energy for the dimer formation reaction is 54 kJ•mol^-1^. Synergistic action of cluster structure formed by sulfonic groups and nanopores in HRF5015 maybe the key factor of high-efficiency catalytic activity and high selectivity. In-situ IR spectra indicate that the intermediate is stable in the reaction process. HRF5015 is environmentally friendly and reusable, which shows good potential in future application.

Keywords

HRF5015, cyclohexanone, self-condensation reaction, dimer, selectivity

File

One file for this dataset

*raw data for the figures (Fig.1, Fig.2, Fig.3, Fig.4 and Fig.8), xlsx. 58KB*

Fig. 1 Effect of temperature on dimer yield

Fig. 2 The relationship between temperature and forward reaction rate

Fig. 3 Effect of catalyst amount on dimer yield

Fig. 4 Catalyst durability test of HRF5015

Fig. 8 In-situ IR spectra of adsorbed cyclohexanone catalyzed by HRF5015
